# The benefits of a comprehensive rehabilitation program in patients diagnosed with spastic quadriplegia

**Published:** 2016

**Authors:** OC Rogoveanu, NC Tuțescu, D Kamal, DO Alexandru, C Kamal, CT Streba, MR Trăistaru

**Affiliations:** * Department of Physical Medicine and Rehabilitation, University of Medicine and Pharmacy of Craiova, Romania; ** Faculty of Nursing and Midwives, University of Medicine and Pharmacy of Craiova, Romania; *** Department of Medical Informatics and Biostatistics, University of Medicine and Pharmacy of Craiova, Romania; **** Department of Family Medicine, University of Medicine and Pharmacy of Craiova, Romania; ***** Department of Research Methodology, University of Medicine and Pharmacy of Craiova, Romania

**Keywords:** quadriplegia spastic, functionality, GMFCS, MACS

## Abstract

Spastic quadriplegia has as an etiopathogenic substrate, a non-progressive brain lesion; however, the clinical manifestations of the disease evolve over time. Children diagnosed with spastic quadriplegia show a variety of symptoms in different areas: sensorimotor, emotional, cognitive, and social. The purpose of this study was to assess the functional status in patients diagnosed with spastic quadriplegia, who followed a complex medical rehabilitation program, during a year, and highlight the importance of using physical and kinetic techniques in improving their status. A total of 10 children diagnosed with spastic quadriplegia were included in the study and the Gross Motor Function Classification System (GMFCS) and manual ability classification system (MACS) were used to evaluate the functionality status of each patient. Every patient was evaluated initially (T1), after six months of program (T2), and after they completed the study. All the children were originally monitored daily, for 5 days per week for a period of one month, then two times a week for a year. A statistically significant difference regarding the modification of the GMFCS and MACS stage was found, which occurred between the first and the third evaluation. The inverse correlation of the statistical significance between the ages of patients and the decrease in GMFCS or MACS stage was highlighted; the younger the patient, the more the scale decreased. A direct link between the gross motor function and the manual ability was noticed. Applying a complex rehabilitation program has proven efficient by improving both the gross motor functionality and the manual ability.

## Introduction

Cerebral palsy is a common cause of occurrence of disability in children. Cerebral palsy brings together a group of diseases that can cause significant changes in posture and motor skills, and can lead to loss of motor autonomy in varying proportions. It is considered that these shortcomings are due to changes made throughout fetal development or in the postpartum period [**1**,**2**].

Depending on the nature of the disorder, three dominant forms of cerebral paralysis can be distinguished: spastic, dyskinetic and ataxic. Depending on the anatomical distribution of the deficit, they are classified in monoplegia, diplegia, hemiplegia, and quadriplegia [**3**].

Quadriplegia affects all four limbs; the spastic form is the most common, generally, the deficit is higher in the upper limbs. Spasticity is present especially in the flexor muscles of the upper limb and lower limb extensor [**1**-**3**].

Destructive damage to neurons in the pyramidal system determines conditioned reflex motor acts deficits. The voluntary conditional reflex movement is the most affected, especially if a high degree of complexity and finesse, such as the use of the hand, is required. The pyramidal system damage does not only permanently abolish the motor conditioned reflexes acts but also some innate reflex motor activities. Thus, cutaneous abdominal reflexes and cremasteric reflexes are affected to the abolition in the pyramidal system lesions [**4**].

Spasticity is one of the dominants of the clinical picture in quadriplegia, and is due to an exaggeration of the tonic and phasic muscle stretch reflex. After a certain time, muscle stiffness may also occur amid this spasticity, seriously aggravating the pre-existing functional deficit. In turn, muscle contracture can be a preliminary stage and a cause that can lead to irreversible muscle retractions [**3**].

The treatment of patients diagnosed with spastic quadriplegia is complex, requires the cooperation of a whole team of specialists, and is based on the full involvement of the family. The therapeutic methods used are medications (anticonvulsants, tranquilizers, neuroleptics, muscle relaxants, neurotrophic), physical therapy, physiotherapy (electrotherapy and thermotherapy), orthopedic and surgical treatment [**5**].

The kinetic treatment consists of using relaxation techniques, massages, posture, passive and active movements, proprioceptive neuromuscular facilitation techniques, exercises for balance, gait rehabilitation, recreational and sports activities and occupational therapy.

The purpose of this study was to assess the functional status in patients diagnosed with spastic quadriplegia, who followed a complex medical rehabilitation for a year, and highlight the importance of using physical techniques in the improvement of their kinetic status.

## Material and methods

A prospective non-experimental (descriptive) observational study, which took place over a year (April 2014 to May 2015), was conducted. A total of 10 children diagnosed with spastic quadriplegia, hospitalized in Filantropia Pediatric Hospital in Craiova, were included. The study received the ethics committee and hospital agreement, all legal guardians of the children included in the study signed a written agreement to participate. All the patients received a comprehensive program that consisted of medical treatment and hygienic-dietary, physical (electro and thermal), physical therapy, massage, occupational therapy.

Gross Motor Function Classification System (GMFCS) was used for the assessment of the motor function. With the help of GMFCS, the presence, amplitude, and force for active movements, particularly walking, were evaluated for each patient [**6**]. The upper limb ability classification manual (MACS) was used to assess functionality [**7**]. MACS is used to assess the child’s overall ability to handle objects from everyday life and does not take into account the functional differences between the two hands.

Each patient was initially evaluated at inclusion (T1), after six months of program (T2), and upon completion of the study. All the children were originally monitored daily, for 5 days per week for a period of one month, then two times a week for a year.

Drug treatment was administered individually (anticonvulsants, tranquilizers, neuroleptics, muscle relaxants, neurotrophic). Physiotherapy treatment methods included both thermotherapy and the use of electrotherapy (galvanizing, low frequency currents, excitatory pulse therapy), individualized for each patient.

The massage techniques used were the sedative massage for the limbs, and facial muscle massage that consisted in light smoothing with a low rhythm and intensity. Massage as a form of introducing kinesiotherapy was also used. Physical therapy was performed daily both individually and in groups [**8**]. Duration physiotherapy sessions averaged 45 minutes, with a break of 5-10 minutes, children not being able to follow and carry out a too long or too complicated kinetic program.

Individual Physical therapy consisted of the following:

1. Posturing in inhibitory reflex positions:

- In dorsal decubitus with a pillow under his head and under the shoulders, upper limbs in indifferent position and lower limbs in extension and abduction,

- Prone with the leg in abduction, at less than 90 degrees, elbow flex, forearm pronation, holding a roll of paper in his hands and legs stretched as far as possible,

- Lateral decubitus; a pillow placed between the legs to promote abduction,

- Sitting with limbs extended, heels resting on the work surface and progressive hip abduction,

- Sitting shortened; child riding position was indicated with the abduction of the thigh; support throughout the foot and varied knee flexion, alternating with the extension.

2. Passive and self-passive mobilization: passive mobilization from reflex-inhibitory positions, slow stretch-reflex, proximal-distal mobilization, analytical exercises, self-passive mobilization such as rolling in bed with the passing of the affected limb slowly over the body midline. Passive exercise duration, which averaged 10 minutes in the physical therapy program.

3. Therapeutic Exercises: Alternating isometric exercises, slow stretching, active relaxation-opposition movements and slow reversals with opposition within Kabat diagonals, concentric isotonic contractions, resistive exercises [**9**], transfer favoring exercises, exercises of quadrupeds, the creeping from a seated position, crossing in orthostatic position, lifting exercises for balance and walking. Breathing exercises, such as deep thoracic-abdominal breathing, were associated to facilitate breathing.

Group physical therapy consisted of physical therapy programs tailored to localize the motor deficit, being applied as adapted games - occupational therapy. Occupational therapy was selected based on the objectives; the format used is shown in **Table 1**.

**Table 1:** Objectives and occupational therapy activities

Objective	Type of activity
Training the capacity to solve problems, visual perception, cognitive, memory and grabbing	The child chooses a box with pieces of plastic letters and forms words with them, placing them on paper, words must be part of a theme and the child has to form a word in 3 minutes.
Training ability to solve problems, cognition, memory, coordination and grabbing	The child chooses a box of cards with numbers and places them on the plate, forming simple equations. Available time: 6 minutes for 3 equations.
Develop coordination, accuracy, picking up	The child must lay 25 beads with a diameter of 1 cm, on a string and then loosen the string, placing the small marbles back in the box. Available time: 10 minutes. Placing wooden disks on sticks and then removing and placing them in the box. Available time - for five rods, 3 discs each, in 10 minutes.
Develop coordination, precision, grabbing and grip strength	From a box of clothespins, removing one pin at a time, pinning them on the edge of the box, and then rearranging them in the box. Available time: 10 clamps in 5 minutes.
Develop coordination, perception and memory	Placing different parts of geometric shapes and colors in a box that presents special places for each piece and putting the pieces back in the box. Available time: 6 pieces in 3 minutes
Develop coordination, perception and mobility	Selecting colorful pieces (plastic cubes with sides of 2 cm) from a basket and putting them on plates according to the color. The basket and plates are spaced 20 cm apart, forcing the child to make wider gestures. Available time: 20 pieces in 10 minutes. Making greeting cards with different themes, performing a range of techniques such as cutting, pasting, edging.

The statistical analysis was performed by using Microsoft Excel (Microsoft Corp., Redmond, WA, USA), together with the XLSTAT add-on for MS Excel (Addinsoft SARL, Paris, France) and IBM SPSS Statistics 20.0 (IBM Corporation, Armonk, NY, USA) for processing the data. 

Because the study involved numerical comparisons between 3 paired data sets that did not have a normal (Gaussian) distribution, the nonparametric Friedman test was primarily used, instead of the ANOVA test, to detect significant differences between the values in the compared data series. The nonparametric Spearman’s rank correlation test was also used to measure the strength of the association between two ranked variables. 

## Results

A total of 10 patients diagnosed with spastic quadriplegia, aged 2 to 11 years, with a mean age of 6.7 years and a standard deviation of 2.75 years, was included in the study.

The group of patients consisted of 6 girls and 4 boys, with a sex ratio girls: boys 1.5: 1. Regarding the residence, 7 patients - 5 girls and 2 boys were from urban areas while 3 patients - one girl and two boys came from a rural residential environment.

**Fig. 1:**
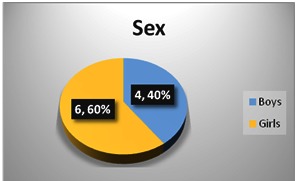
Distribution according to sex in the studied group

**Fig. 2:**
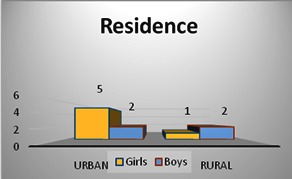
Residence of studied group

**Fig. 3:**
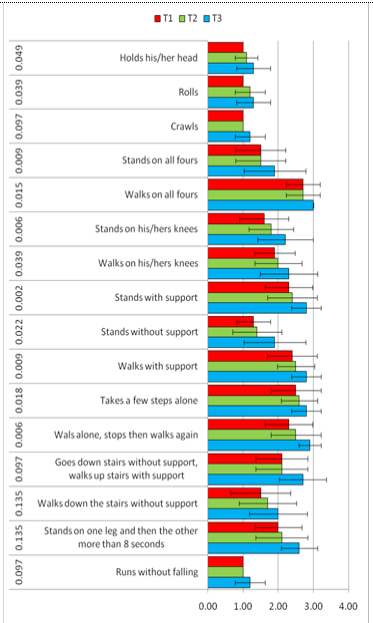
Differences in the main parameters followed by GMFCS

The nonparametric Friedman test was used to compare parameter values observed for the gross motor function testing, in the 3 evaluation stages. Significant differences occurring between T1 and the other two points are identified in **Table 2** and illustrated in **Fig. 3**.

**Table 2:** The differences of parameters pursued in GMCFS

T1	T2	T3	p Friedman	Significance
1.00 ± 0.00	1.10 ± 0.32	1.30 ± 0.48	0.049	S
1.00 ± 0.00	1.20 ± 0.42	1.30 ± 0.48	0.039	S
1.00 ± 0.00	1.00 ± 0.00	1.20 ± 0.42	0.097	NS
1.50 ± 0.71	1.50 ± 0.71	1.90 ± 0.88	0.009	S
2.70 ± 0.48	2.70 ± 0.48	3.00 ± 0.00	0.015	S
1.60 ± 0.70	1.80 ± 0.63	2.20 ± 0.79	0.006	S
1.90 ± 0.57	2.00 ± 0.67	2.30 ± 0.82	0.039	S
2.30 ± 0.67	2.40 ± 0.70	2.80 ± 0.42	0.002	S
1.30 ± 0.48	1.40 ± 0.70	1.90 ± 0.88	0.022	S
2.40 ± 0.70	2.50 ± 0.53	2.80 ± 0.42	0.009	S
2.50 ± 0.71	2.60 ± 0.52	2.80 ± 0.42	0.018	S
2.30 ± 0.67	2.50 ± 0.71	2.90 ± 0.32	0.006	S
2.10 ± 0.74	2.10 ± 0.74	2.70 ± 0.67	0.097	NS
1.50 ± 0.85	1.70 ± 0.82	2.00 ± 0.82	0.135	NS
2.00 ± 0.67	2.10 ± 0.74	2.60 ± 0.52	0.135	NS
1.00 ± 0.00	1.00 ± 0.00	1.20 ± 0.42	0.097	NS

A significant difference in the change of GMFCS stage, which occurred between the first and the third evaluation, p-value (two-tailed) = 0.000562703, was found.

 There was a significant difference in the change that occurred between the first MACS stage and the third evaluation, p-value (two-tailed) = 0.000819232.

**Fig. 4:**
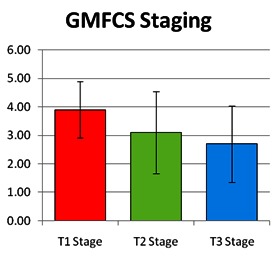
The evolution of gross motor skills functionality

**Fig. 5:**
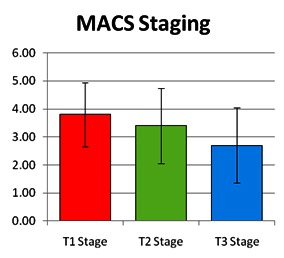
The evolution of manual abilities

By calculating the Spearman correlation coefficient, statistically significant inverse correlations were found between the decrease in ages and the GMFCS stage (Spearman rho = -0.457) and decreased stage MACS (Spearman rho = - 0.365), the decrease being more important as the patient’s age was lower.

**Fig. 6:**
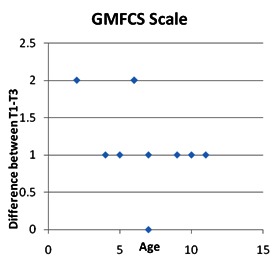
Correlations between patient age and GMFCS stage

**Fig. 7:**
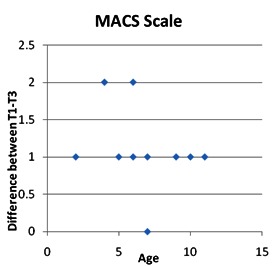
Correlations between patient age and MACS stage

A statistically significant correlation was also found between GMFCS and MACS score between the first and the third evaluation, the Spearman rho correlation coefficient being 0.501 (p <0.05).

## Discussions

Cerebral palsy is one of the most common causes of occurrence of disability in children, with a prevalence of 2-2.5 cases/ children [**2**]. The spastic form is found at a rate of 75-80% [**3**]. Impaired motor activity is the main feature in patients with cerebral palsy and is frequently accompanied by impaired sensory perception, cognition, communication, and behavior.

Although in most cases, spastic quadriplegia’s etiopathogenic substrate is a non-progressive brain damage, clinical manifestations of the disease evolve over time [**5**,**10**,**11**]. Patients often associated with spastic quadriplegia associate different types of pathologies, comorbidities and those are usually the ones that favor dysfunctionality in these children.

In addition to improving the physical, mental, and social areas, the maintaining and preserving of a good functionality is a goal in patients diagnosed with spastic quadriplegia. The most frequently encountered disabilities in these patients are self-care related and gross motor function motility [**3**].

The complex program of rehabilitation that patients in the study group followed was individualized according to the initial evolutionary stage, comorbidities, taking into account factors like psychiatric disorders, and the degree of involvement of the family.

All the therapeutic means used throughout the program complied with the no pain criteria, the daily program duration was long enough to accomplish the goals, but without getting tiresome or boring for the children, as they were not able to follow and carry out a program that was too long or too complicated.

Determining the motor and functional status in patients diagnosed with spastic quadriplegia is extremely important for the assessment of the effectiveness of the therapeutic methods in recovery programs.

In order to determine the degree of the gross motor function, GMFCS classification system was used; for the motor function and the manual skills were evaluated by using the MACS classification system, both proving their effectiveness in a variety of studies and clinical trials [**8**,**12**-**14**].

A total of 10 patients diagnosed with spastic quadriplegia, aged 2 to 11 years, with a mean age of 6.7 years and a standard deviation of 2.75 years were included in the study.

It was shown that a coarse motor function deteriorates with age in patients diagnosed with spastic quadriplegia. It is believed that the gross motor activity reaches a maximum around the age of 6-7 [**11**] and then decreases gradually. Several studies have demonstrated that the damage to the functionality occurs mainly in children diagnosed with spastic quadriplegia, after 9 years of age [**15**-**20**].

The group of patients consisted of 6 girls and 4 boys, with a sex ratio girl/ boy of 1.5: 1. Regarding the residence, 7 patients - 5 girls and 2 boys were from urban areas, while 3 patients - one girl and two boys had a rural residential environment.

Due to the difficulty in applying steady recovery programs, in some studies, patients from rural areas showed a significant long-term improvement in the functional status. Our study was not influenced during the course of the program because we benefited from the support and involvement of the families who have complied with the pace (5 sessions/ week) and daily duration of the proposed program [**21**].

Nonparametric Friedman test was used to compare the parameter values observed for the gross motor function testing at the 3 evaluation points. A significant difference between T1 and the other two evaluation points was highlighted.

The effectiveness of the complex rehabilitation program followed by the group of studied patients was demonstrated by parameter changes of the gross motor function classification system. Our data correlated with those reported in the literature [**22**].

A significant difference in the modification, for the better, of the gross motor function, which occurred between the first and the third evaluation, p-value (two-tailed) = 0.000562703, was found especially in patients who were included at baseline in stages II and III of the gross motor function.

After applying a comprehensive rehabilitation program for patients diagnosed with spastic quadriplegia, there was a significant improvement in the coarse motility in those who had a better gross motor functionality score [**5**].

There was a significant difference in terms of modification of manual ability between the first and the third evaluation, p-value (two-tailed) = 0.000819232.

Both classification systems, GMFCS and MACS, have proven useful in assessing functionality in patients diagnosed with spastic quadriplegia, being extremely useful in determining the value of applying rehabilitation programs [**23**].

By calculating the Spearman correlation coefficient, an inverse correlation of statistical significance was identified between the ages of patients and the coarse motor function (Spearman rho = -0.457) and the manual ability (Spearman rho = - 0.365), as the patient’s age was lower, the favorable development was more important.

In patients older than 10 years with a lower original score for coarse functionality, regardless of the duration and intensity of the applied program, we did not obtain satisfactory results [**5**].

The data contained in the literature showed that setting up a complex program rehabilitation as early as possible after diagnosis and continuing it for as long as possible is fundamental in recovering functionality in patients with spastic quadriplegia [**9**,**21**].

A statistically significant correlation was found between the gross motor functioning scores differences and the manual ability between the first and third evaluation, the Spearman rho correlation coefficient being of 0.501 (p <0.05). A direct link was observed between the gross motor function and the manual ability. A significant improvement in the MACS score was observed in patients with a better GMFCS score.

The gross mobility classification system score was used as a predictor of the functional status in other studies, where it was noted that in patients with a better score, the rehabilitation methods led to a better recovery of the motor deficit [**22**].

In a study in which it was proposed to establish a relationship between the two types of functionality (motor coarse and the manual ability) in patients diagnosed with different types of cerebral palsy, significant statistical correlations have been demonstrated only in patients with spastic quadriplegia [**24**]. Improving the gross motor function classified by using the gross motor functional score correlated with the ability evaluated by the manual classification system for manual ability in other studies [**25**]. The individualization of the rehabilitation program was one of the reasons for a favorable gross motor functionality and manual ability for patients in the studied group. 

## Conclusion

Applying a complex rehabilitation program has proven its worth by improving the gross motor functionality and the manual ability, being a direct link between their development stages. The initiation of a rehabilitation program as early as possible after diagnosis is imperative, as it was highlighted by the inverse correlation of the statistical significance between the ages of patients and the improvement of the gross motor functionality and manual ability. The individualization of the rehabilitation program and the family involvement are essential in the functionality of the favorable development of the gross motor and the manual ability in children with spastic quadriplegia.

**Conflict of interests**

The authors declare that they have no conflict of interests.

**Contribution note**

All authors have contributed equally to this work.
